# Effect of Moderate Consumption of Different Phenolic-Content Beers on the Human Gut Microbiota Composition: A Randomized Crossover Trial

**DOI:** 10.3390/antiox11040696

**Published:** 2022-03-31

**Authors:** José Ignacio Martínez-Montoro, Mar Quesada-Molina, Carolina Gutiérrez-Repiso, Patricia Ruiz-Limón, Alba Subiri-Verdugo, Francisco J. Tinahones, Isabel Moreno-Indias

**Affiliations:** 1 Department of Endocrinology and Nutrition, Virgen de la Victoria University Hospital, Instituto de Investigación Biomédica de Málaga (IBIMA), 29010 Málaga, Spain; joseimartinezmontoro@gmail.com (J.I.M.-M.); marquesadamolina@gmail.com (M.Q.-M.); gutierrezrepiso@gmail.com (C.G.-R.); patriciaruizlimon@ibima.eu (P.R.-L.); alb.sub@gmail.com (A.S.-V.); isabel.moreno@ibima.eu (I.M.-I.); 2 Faculty of Medicine, University of Málaga, 29010 Málaga, Spain; 3 Centro de Investigación Biomédica en Red de la Fisiopatología de la Obesidad y Nutrición (CIBEROBN CB06/003), Instituto de Salud Carlos III, 28029 Madrid, Spain

**Keywords:** beer, polyphenols, antioxidants, gut microbiota

## Abstract

The moderate consumption of beer has been associated with positive effects on health, and these benefits are driven, in part, by the antioxidant properties of phenolic compounds found in this beverage. However, the potential impact of beer polyphenols on the human gut microbiome and their consequences are yet to be elucidated. In this study, our aim was to evaluate the effect of three different phenolic-content beers on the gut microbiome and the potential role of the induced shifts in the antioxidant capacity of beer polyphenols. In total, 20 subjects (10 healthy volunteers and 10 individuals with metabolic syndrome) were randomly assigned in a crossover design to consume each of the different beers (alcohol-free, lager or dark beer) during a 2-week intervention. Significant changes in the relative abundance of *Streptococcaceae* and *Streptococcus* were found after beer consumption. An increased abundance of *Streptococcaceae* and *Streptococcus* was observed after the consumption of dark beer, with no detected differences between baseline and alcohol-free/lager beer intervention. Moreover, some of the detected differences appeared to be related to the metabolic status. Finally, a decrease in porphyrin metabolism and heme biosynthesis was found after the intervention, especially after the consumption of dark beer. These results show that the antioxidant capacity of beer polyphenols may induce positive shifts in gut microbiota composition, and some of the observed changes may also boost the antioxidant capacity of these compounds.

## 1. Introduction

In recent decades, beer has become the most important source of per capita alcohol consumption in the United States and several European countries [1,2]. Despite the fact that alcohol abuse has been demonstrated to be associated with substantial morbidity and mortality [3], moderate alcohol consumption has been related to some beneficial effects on cardiovascular and metabolic health [4]. In line with this, moderate beer consumption could also be related to cardioprotective benefits and positive overall health outcomes [5,6].

The potential mechanisms through which beer and other fermented beverages mediate these health benefits may be linked to the anti-inflammatory and antioxidant properties conferred by their high phenolic compounds content [7]. Thus, beer polyphenols from malt and hop may contribute to the protection of the cardiovascular and endocrine system, among other functions [8]. However, it is important to note that the complex pathways involved in beer-related actions are not fully understood, and additional routes are yet to be elucidated.

In recent years, the gut microbiota has emerged as a crucial player in human health and disease [9]. Composed of trillions of microorganisms, this complex community performs essential functions in the host, including the modulation of different inflammatory, immune and hormonal axes [10]. Remarkably, an extensive body of evidence shows that several chronic diseases, including metabolic disorders and some types of cancers, may develop when the fragile balance of intestinal homeostasis is disrupted, leading to dysbiosis [11]. In this regard, environmental factors, such as dietary components, are able to induce significant shifts in gut microbiota composition, a fact that could have a major role in the pathophysiology and prevention of a number of disorders. 

Previous studies have described several interactions between beer polyphenols and the gut microbiome, resulting in potential health benefits, although this evidence mainly comes from in vitro testing [12,13]. In animal models, ferulic acid (the most abundant polyphenol in beer) has been reported to increase bacterial richness and diversity and to stimulate the growth of butyrate- and propionate-producing strains [14,15]. Tetrahydro *iso*-alpha acids from hops decreased plasma levels of lipopolysaccharide and pro-inflammatory cytokines and increased the expression of tight junction proteins and the anti-inflammatory cytokine interleukin-10, resulting in improved glucose homeostasis and body weight reduction in high-fat-diet-fed mice [16]. On the other hand, there are a limited number of clinical studies assessing the role of beer/beer polyphenols in gut microbiota. In an observational study including healthy volunteers (abstainers and moderate beer consumers), butyric acid concentrations, which correlated with *Pseudobutyrvibrio*, were increased in moderate consumers of beer [17]. In a study conducted with 35 healthy volunteers, alcoholic and non-alcoholic beer increased the abundance of *Bacteroidetes* and decreased the growth of *Firmicutes*, and the latter was associated with an enrichment of gut microbiota diversity; these results were postulated to be due, in part, to the biological activity of polyphenols and phenolic acids from beer [18]. Recently, in a pilot trial, we reported that alcohol-free beer enriched with isomaltulose produced several changes in the gut microbiota in subjects with type 2 diabetes mellitus (T2DM) and overweight/obesity and decreased glucose levels and homeostatic model of insulin resistance (HOMA-IR) [19].

In light of the above, although the positive impact of the moderate consumption of beer on health may be driven by major changes in the gut microbiota induced by phenolic compounds and their antioxidant properties, further studies in humans are needed to identify specific changes in gut microbiota composition and their potential effects. Hence, in this trial, our aim was to evaluate the influence of the moderate consumption of three different beers with distinct polyphenol concentrations (alcohol-free beer—low polyphenol content; lager beer—intermediate polyphenol content; and dark beer—high polyphenol content) on gut microbiota composition in healthy volunteers and patients with metabolic syndrome. 

## 2. Materials and Methods

### 2.1. Study Design and Participants

**Figure 1:**
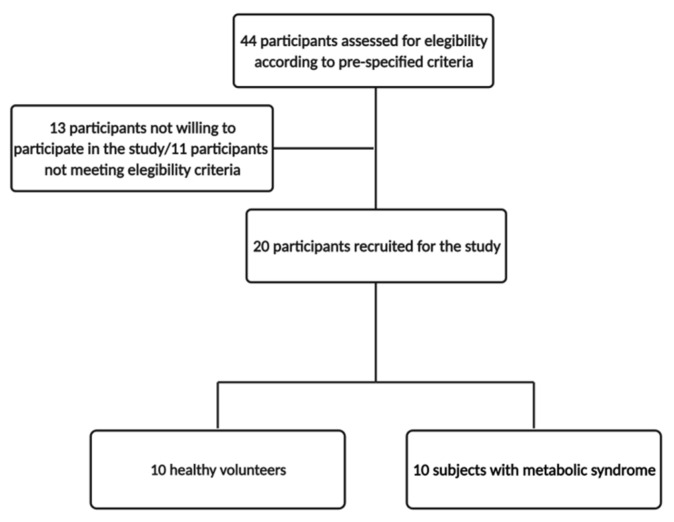
Study course flowchart.

This study was a randomized open-label crossover trial (ClinicalTrials.gov Identifier NCT05300165) conducted at Virgen de la Victoria University Hospital (Málaga, Spain). Adult individuals aged 30–60 years old were included in this study. Exclusion criteria comprised the presence of morbid obesity (body mass index ≥ 40 kg/m^2^), alcohol consumption over 30 g/20 g per day for men and women, respectively, and the intake of antibiotics, prebiotics, probiotics vitamin supplementation or any other medication that could influence the gut microbiota composition. During the study intervention, participants were not allowed to intake alcoholic beverages different from those provided or vitamin supplements/probiotics/prebiotics/antibiotics/other drugs with potential effects on the microbiome. Volunteers were divided in two groups: healthy volunteers (all of them presenting a BMI <30 kg/m^2^) and subjects with metabolic syndrome (according to the National Cholesterol Education Program Adult Treatment Panel III [20], presenting three or more of the following criteria: waist circumference > 102 cm in men/>88 cm in women; blood pressure > 130/85 mmHg; fasting triglyceride-TG-levels > 150 mg/dL; fasting high-density lipoprotein < 40 mg/dL in men/<50 mg/dL in women; and fasting glucose levels > 110 mg/dL). The study course flowchart is summarized in Figure 1.

### 2.2. Study Intervention

After a 2-week washout period, participants were randomly assigned in a crossover design to determine the order in which they would receive each of the three interventions: (1) alcohol-free beer (low polyphenol content—12.2 mg/100 mL); (2) lager beer (intermediate polyphenol content—27.83 mg/100 mL; 4.2% alcohol by volume); and (3) dark beer (high polyphenol content—41.6 mg/100 mL; 4.5% alcohol by volume); polyphenol content was estimated using the public database Phenol-Explorer [21]. Each intervention consisted of 2 weeks consuming one bottle (33 cl) of the pertinent beer daily. Anthropometric, biochemical and dietary assessments were performed after washout and at the end of each intervention. Feces samples were also collected at the end of each intervention and were stored at −80 °C until the end of the study. 

### 2.3. Sample Processing and Biochemical Evaluation

Blood samples were collected after 10 h of fasting. Biochemical parameters were measured using standard enzymatic methods. Glycosylated hemoglobin was determined using reverse-phase cationic chromatography and double wave-length colorimetry quantification (ADAMS A1c HA-810, Arkray Factory, Shiga, Japan). C-Reactive protein was measured using nephelometry (MMAGE-Immunochemistry Systems, Beckman Coulter, Brea, CA, USA). HOMA-IR was estimated using the formula glucose (mg dL^−^^1^) × plasma insulin (μU mL^−^^1^)/405. Urine and fecal samples were collected and stored at −80 °C until the end of the study.

### 2.4. Gut Microbiome Analysis

DNA extraction from tools was performed using the Maxwell^®^ RSC blood kit, following the manufacturer’s instructions. DNA concentration was determined via absorbance at 260 nm, and DNA purity was estimated with a Nanodrop spectrophotometer (Nanodrop Technologies, Wilmington, DE, USA). The Metagenomics Kit (Thermo Fischer Scientific, Waltham, MA, USA, EE.UU.), consisting of primer pools to amplify multiple variable regions of the 16S rRNA (V2-4-8 and V3-6, 7-9), was used. These regions were selectively amplified via polymerase chain reaction—PCR (10 min at 95 °C, followed by 18–25 cycles of 30 s at 95 °C, 30 s at 58 °C, 20 s at 72 °C and, finally, 7 min at 72 °C). After the visualization of PCR products on agarose gel, they were purified using Agentcout^®^ AMPure^®^ XP technology (Beckman Coulter, Indianapolis, IN, USA). The resulting DNA was quantified via fluorimetry using a Qubit™ dsDNA BR Assay kit (Thermo Fischer Scientific, Wlatham, MA, USA). Libraries from stool samples were built with the Ion Plus Fragment Library kit (Thermo Fischer Scientific, Waltham, MA, USA) and barcoded with the Ion Xpress™ Barcode Adapters kit (Thermo Fischer Scientific, Waltham, MA, USA). Barcoded libraries were pooled and templated using the automated Ion Chef (Thermo Fischer Scientific, Waltham, MA, USA) with Ion Sequencing 510 & 520 & 530TM sequencing Kit-Chef reactives (Thermo Fischer Scientific, Waltham, MA, USA) and the Ion 530TM Chip Kit, followed by sequencing using the Ion Torrent S5™ (Thermo Fischer Scientific, Waltham, MA, USA).

### 2.5. Ethics

The study was conducted in accordance with the Declaration of Helsinki and approved by the Ethics Committee of Virgen de la Vitoria University Hospital with the reference ID BEEROTA18 “Role of gut microbiome in the health benefits associated to the moderate consumption of beer” on the 27 September 2018. Informed consent was obtained from all subjects involved in the study. 

### 2.6. Statistical Analysis

Bioinformatic analysis was performed using the open source tool QIIME 2 2019.10 software [22]. Quality sequences were obtained and demultiplexed using the Ion Reporter Software version 4.0 (Thermo Fisher Scientific Inc., Wlatham, MA, USA) using the “fastq creator” plugin. Amplicons Sequence Variants (ASVs) were calculated using DADA2 [23]. Representative ASVs were used to build the phylogenetic tree. Alpha and beta diversities were also assessed with QIIME 2 (“diversity” plugin). Weighted UniFrac distance was used for the assessment of beta diversity [24]. VSEARCH and the Greengenenes database version 13_8 clustered at 97% identity were used for the taxonomic classification of the features [25]. Statistical software Microbiome Analyst, through the web microbiomeanalyst.ca [26] and DESeq2 [27], was used to perform the differential analysis between experimental groups and bacterial relative abundances (at the levels of phylum, family and genus). Groups differences in terms of alpha diversity were assessed using the Kruskal–Wallis test, whereas beta diversity was assessed using PERMANOVA. Longitudinal analysis of gut microbiome dynamics was assessed via QIIME2 (“longitudinal” plugin). PICRUSt2 was used to predict the functional profile of microbial communities [28] within QIIME2. MetaCyc pathways [29] were normalized within QIIME2 and further analyzed with STAMP [30]. p values were corrected for multiple comparisons using the Benjamini–Hochberg method and reported as q-values where appropriate. Results were assumed as statistically significant when *p* < 0.05 and q < 0.20, although results with a *p* < 0.05 and q > 0.20 were also reported because of their informative value. 

Anthropometric and biochemical parameters, as well as the results related to dietary assessment, were analyzed using IBM SPSS Statistics version 25.0 (IBM Corporation, Armonk, NY, USA). The Shapiro–Wilk test was used to assess the normal distribution of the variables in our population. A Friedman test was used to compare characteristics between groups. 

## 3. Results

### 3.1. Study Population

A total of 20 participants (10 healthy volunteers and 10 subjects with metabolic syndrome) aged 30–60 years old were included in this study. Figure 1 shows the study flowchart. 

### 3.2. Dietary Assessment

First of all, dietary records were obtained from participants during the study in order to determine potential differences in dietary habits that would explain gut microbiome changes beyond the study interventions (Supplemental Table S1). No significant differences were found with regard to total energy, lipid, protein, carbohydrate, simple sugars or fiber intake among the three interventions in the whole population. However, the consumption of simple sugars was significantly higher (*p* = 0.006) in healthy volunteers compared with subjects with metabolic syndrome. 

### 3.3. Clinical, Anthropometric and Biochemical Parameters

Table 1 summarizes the basal characteristics of the study population obtained after the washout period and at the end of each intervention (dark/lager/alcohol-free beer). The study population is separated by metabolic status (i.e., healthy volunteers and patients with metabolic syndrome). Interestingly, the main differences observed in the study population after the different interventions were only observed in the metabolic syndrome group. Hence, uric acid levels significantly increased (within normal serum levels), whereas glycosylated hemoglobin (HbA1c, %) modestly decreased after the three interventions in this group. On the other hand, HDL-cholesterol levels modestlyincreased after the intake of dark/lager beer in healthy volunteers. 

### 3.4. Gut Microbiota

#### 3.4.1. Diversity

No differences were found in alpha diversity parameters between groups (baseline, dark beer, lager beer and alcohol-free beer): richness (observed features; *p* = 0.75); evenness (Pielou index; *p* = 0.41); diversity (Shannon index; *p* = 0.55) or phylogenetic diversity (PD; *p* = 0.85).

**Figure 2:**
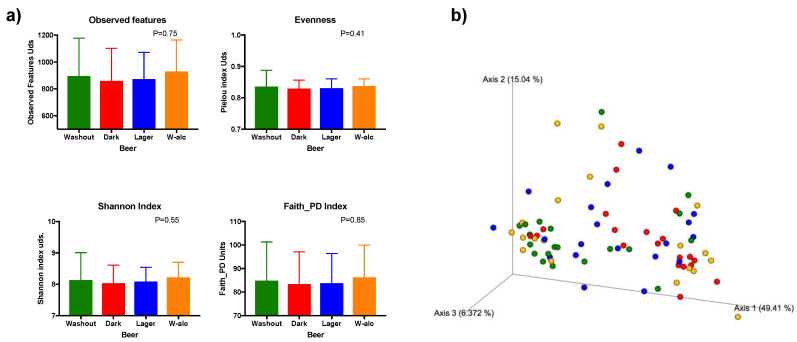
Gut microbiota diversities at the different beer periods. (**a**) Alpha diversity indexes: observed features, evenness, Shannon index and Faith_PD index; values are presented as mean ± SD (standard deviation). (**b**) Principal coordinates analysis plot of weighted Unifrac distance of fecal samples collected at the end of each beer period. Baseline (washout—green dots) and at the end of each intervention period (dark beer—red dots; lager beer—blue dots; alcohol-free beer—yellow dots).

Similarly, there were no differences between groups with regard to beta diversity—Figure 2 (unweighted Unifrac distance, *p* = 0.99; weighted Unifrac distance, *p* = 0.054). However, nearly significant weighted Unifrac distance was achieved (*p* = 0.054) mainly due to differences between baseline and dark beer intervention (*p* = 0.01, q = 0.06).

#### 3.4.2. Bacterial Relative Abundances

**Figure 3:**
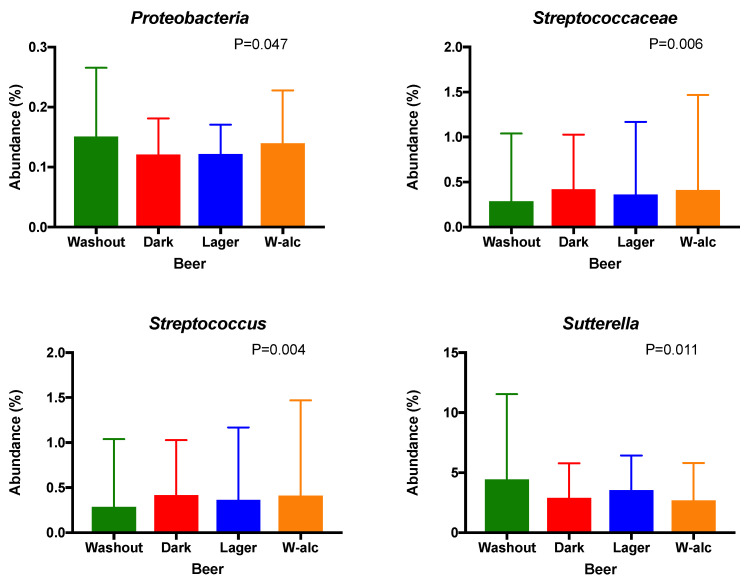
Taxa that significantly changed between the different intervention periods at different taxonomic levels: phylum, family and genus (DesSeq2 test; *p* < 0.05). Values are presented as mean ± SD (standard deviation).

At the family and genus levels, *Streptococcaceae* and *Streptococcus,* respectively, were found to be different between groups (*p* = 0.006, q = 0.174; *p* = 0.004, q = 0.160, respectively, Figure 3). Additionally, *Sutterella* genus reached statistical significance after multiple testing corrections (*p* = 0.011, q = 0.196, Figure 3). No significant differences were observed at phylum level between groups, although *Proteobacteria* was nearly significant (no differences after correcting for multiple comparisons *p* = 0.047, q = 0.333). 

#### 3.4.3. Gut Microbiota Changes According to the Type of Beer

In order to deepen our understanding of the changes observed with every type of beer, we analyzed changes in each beer intervention with respect to the washout period. 

After the dark beer intervention period, although no changes were found in alpha diversity (observed features, *p* = 0.55; evenness, *p* = 0.14; Shannon index, *p* = 0.27; Faith_PD, *p* = 0.61) and the qualitative beta diversity assessment (unweighted Unifrac, *p* = 0.961), a significant difference was found between the microbiota population at baseline and dark beer (weighted Unifrac, *p* = 0.006). Going further, significant changes were also found at the family *Streptococcaceae* (*p* = 0.002, q = 0.069) and in its genus *Streptococcus* (*p* = 0.001, q = 0.045) levels (Supplemental Figure S1a). 

Regarding lager beer consumption, no changes were observed related to alpha diversity (observed features, *p* = 0.617; evenness, *p* = 0.267; Shannon index, *p* = 0.318; PD, *p* = 0.665) or beta diversity (unweighted Unifrac, *p* = 0.939; weighted Unifrac, *p* = 0.126). Regarding changes in particular taxa, although no further differences were obtained when *p*-values were corrected by multiple comparisons, it is worth mentioning that the phylum *Verucomicrobia* (*p* = 0.041, q = 0.286), its family *Verrucomicrobiaceae* (*p* = 0.038, q = 0.999) and the genera *Blautia*, *Lachnospira* and *Akkermansia* showed *p*-value < 0.05 (*p* = 0.010, *p* = 0.044; *p* = 0.039, q = 0.487; q = 0.344, q = 0.487) (Supplemental Figure S1b). 

Similarly, the alcohol-free beer consumption resulted in no differences in alpha diversity (observed features, *p* = 0.895; evenness, *p* = 0.715; Shannon index, *p* = 0.530; PD, *p* = 0.942) or beta diversity (unweighted Unifrac, *p* = 0.997; weighted Unifrac, *p* = 0.184). Regarding changes in particular taxa, the phylum *Verrucomicrobia* (*p* = 0.012, q = 0.088) and the genus *Ruminococcus* (*p* = 0.023, q = 0.705) showed *p*-values < 0.05 (Supplemental Figure S1c).

#### 3.4.4. Metabolic Pathways

**Figure 4:**
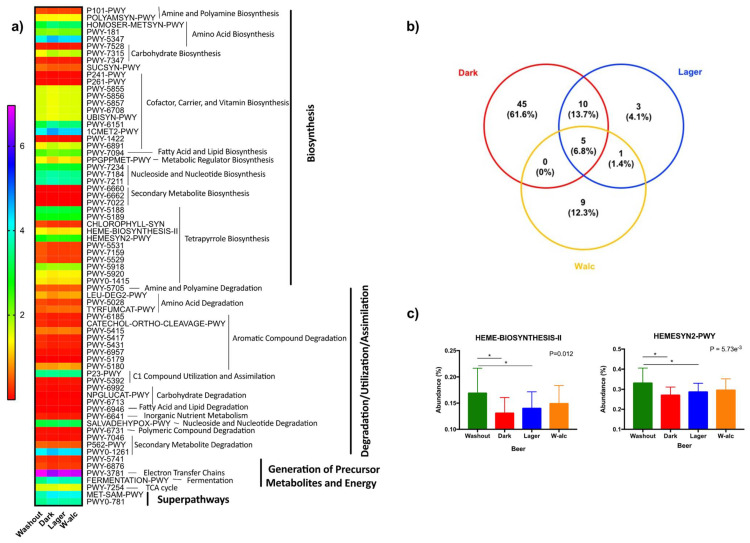
Predictive metabolic pathways by PICRUst2. (**a**) Differentially abundant Metacyc pathways identified after the intervention periods (mean values are presented. Supplemental Table S2 shows each pathway names). (**b**) Venn diagram of the Metacyc pathways that differed between the washout and each beer period (Supplemental Table S3 shows pathways shared and not shared by the different beer periods). (**c**) Porphyrin metabolism and heme biosynthesis routes in the washout period and after the consumption of the different types of beers. Values are presented as mean ± SD (standard deviation). * Statistically significant differences between groups (*p* < 0.05).

Biochemical pathways were inferred through PICRUSt2. Figure 4 shows Metacyc pathways that differed between beers periods. Although there were minimal changes, dark beer seemed to behave in a different way with respect to the other periods. Upon further analysis focusing on those pathways changing with respect to the washout period, the Venn diagram shows those pathways shared by the different beers. Again, the dark beer showed the highest rate of change. Interestingly, pathways related to Porphyrin Compound Biosynthesis seem to be more affected by beer, and especially by alcohol beer consumption. Indeed, the analysis showed a significant decrease in porphyrin metabolism and heme biosynthesis after beer consumption, with a deeper decrease after the consumption of dark beer (Figure 4). 

#### 3.4.5. Microbiota Results in Relation with the Metabolic Status of the Volunteers

**Figure 5:**
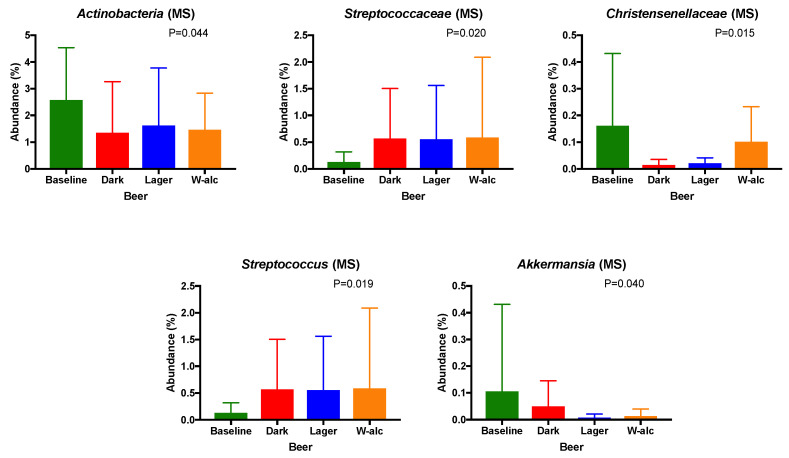
Taxa that changed between the different intervention periods at different taxonomic levels: phylum, family and genus (DesSeq2 test; *p* < 0.05) within the volunteers with metabolic syndrome. Values are presented as mean ± SD (standard deviation).

In line with the anthropometric and biochemical results, according to subgroups (control and patients with metabolic syndrome), the main changes were observed in patients with metabolic syndrome. Indeed, no significant differences were observed between baseline and dark beer/lager beer/alcohol-free beer interventions in healthy volunteers. On the other hand, subjects with metabolic syndrome tended to present lower relative abundances of *Actinobacteria* and *Christensenellaceae* (*p* = 0.044, q = 0.293; *p* = 0.015, q = 0.293; respectively, Figure 5), and higher relative abundances of *Streptococcaceae* and *Streptococcus* after beer consumption (*p* = 0.020, q = 0.293; *p* = 0.019, q = 0.617; respectively, Figure 5), while *Akkermansia* generally reduced its relative abundance with beer consumption (*p* = 0.040, q = 0.637, Figure 5). These results, although worth mentioning, should be taken cautiously, as they did not overcome the FDR multiple correction threshold.

## 4. Discussion

In this human randomized trial, we demonstrate that the chronic moderate consumption of beer induces several changes in gut microbiota composition. Moreover, these changes are related to the type of beer consumed, as they are more evident after the consumption of dark beer (high polyphenol content). Additionally, some of the observed shifts in the relative abundance of different gut bacteria after the consumption of beer appear to be related to the metabolic status of the host. 

Several reports have indicated that beer consumption affects gut microbiota diversity. In the current study, we did not observe a significant change in the alpha diversity of the populations. Other studies, such as the one of Hernández-Quiroz, reported an increase in richness and biodiversity with an alcohol-free beer [18], but we did not observe these results, maybe because of the smaller period of time used for alcohol-free beer. Interestingly, we observed a change in the whole microbial community profile, indicated by the variation in beta diversity between the different beer periods, especially related to the dark beer period. Moreover, this tendency was confirmed when comparing dark beer with baseline gut microbial characteristics. Others did not find differences in the gut microbiota whole population related to beer consumption in general [17], while others found these with an alcohol-free regular beer, but not with a regular beer [18], indicating the possible interference of alcohol. In our study, even with a reduced consumption period of each beer, we observed that the type of beer is relevant for the interaction with gut microbiota. Previously, others studied two types of beer: with or without alcohol. In this regard, Hernández-Quiroz et al. showed that the moderate consumption of non-alcoholic beer had positive effects on human health and gut microbiota by the antioxidant activity of phenolic acids, whereas the presence of alcohol in beer interfered with these outcomes [18]. In this manner, phenolic content has been previously described as an important marker of metabolic health [31]. Therefore, the phenolic content of beer could cause an interaction. In fact, under our current design, dark beer is the one with the highest phenolic content. In this regard, phenols and melanoidins, abundant in dark beer [32], have been demonstrated to be powerful natural antioxidant agents that protect against damage induced by reactive oxygen species (ROS), and these effects seem to be more intense in dark beer, a fact that could have a strong impact on gut microbiota diversity [7]. Although it is difficult to unravel the proportion of our results that can be attributed to phenolic compounds or alcohol, our findings suggest that alcohol content in beers cannot be the only determinant conditioning gut microbial changes after beer consumption, since different results were observed after lager and dark beer intervention, which present almost identical alcohol contents (4.2% and 4.5%, respectively). Thus, additional beer components, such as polyphenols, may have a role in gut microbiota composition, as previously discussed. On the other hand, although it is difficult to unravel the role of different sugar consumptions between groups in the results, given the fact that sugar consumption was not different among washout and intervention periods in the pertinent groups, we consider that this fact did not have a significant impact on beer-related outcomes. 

Once we recognized some changes related to the consumption of different beers, we found that the relative abundance of *Streptococcus* is different between groups, especially due to the consumption of dark beer, which provoked a significant increase in the abundance of this genus. In a context of alcohol overconsumption triggering gut dysbiosis [33], *Streptococcus* was demonstrated as the best bacterium to predict the severity of liver injury in alcoholic liver disease [34]. However, the amount of alcohol ingested in this trial, one bottle per day, discards this option. Previous research has shown that the interaction between polyphenolic compounds, such as gallic acid and catechins, which are present in beer, and some bacterial strains (e.g., *Streptococcus thermophilus*) may potentiate the antioxidant effects of polyphenols [35]. Additionally, melanoidins from beer can be used by some gut bacteria, such as *Streptococcus intermedius*, to produce the antioxidant equol [36]. Modest changes in glycosylated hemoglobin and improved glucose homeostasis observed in our study after beer consumption may also be related to this genus, as demonstrated in previous studies [18]. Additionally, the decline in *Sutterella* relative abundance could have influenced these results because of its mild pro-inflammatory capacities [37]. 

Analyzing each beer period further, the phylum *Verrucomicrobia* was reduced after the lager and non-alcoholic beers. Moreover, this trend was followed in the lager period for its genus *Verrucomicrobiaceae* and its genus *Akkermansia*. In the dark beer period, this trend was not observed. This genus has been extensively studied, and its health-promoting effects are widely recognized [38]. Interestingly, *Akkermansia muciniphila* has been associated with reduced damage induced by gluco- and lipo-toxicity, oxidative stress and inflammation [39]. Dark beer, through its polyphenol-rich content, could mitigate the decline in these bacteria.

The relative abundance of *Lachnospira* was found to be increased after the lager period, in line with a previous study that reported an increase in *Lachnospira* as a consequence of moderate beer consumption [17]. This genus has the potential to produce short-chain fatty acids [40], and in fact, a rise in *Lachnospiraceae* has been related with some of the benefits associated with the consumption of a Mediterranean diet [41]. However, *Blautia* (also a *Lachnospiraceae* genus) reduced its levels in the lager period. This genus has been related with multiple proinflammatory diseases such as non-alcoholic steatohepatitis (NASH) or type 1 diabetes [42].

Interestingly, attending to the metabolic status of the participants, different results were observed after beer consumption. On the one hand, no significant changes in gut microbiota composition were found in healthy subjects after the intervention. Conversely, there was a tendency towards significant shifts among individuals with metabolic syndrome. Thus, in a similar fashion as the general group, a tendency to the increase in the relative abundance of *Streptococcaceae* and its genus *Streptococcus*, and a tendency to the decrease in *Akkermansia* (especially after the consumption of lager beer, but not dark beer) was noticed in this subgroup. In view of these results and considering the aforementioned consequences of these shifts, it could be speculated that the potential beneficial effects of beer polyphenols, including their well-known antioxidant activity, may be stronger in the presence of characteristics associated with gut dysbiosis (i.e., metabolic disturbances), and the antioxidant properties of these compounds may be mediated by the restoration of eubiosis, which could in turn confer direct protection against oxidative stress. However, further research is needed to confirm this hypothesis. 

It is noteworthy that novel strategies for a better understanding of the relationship between gut microbiota and the host are emerging. Accordingly, “pantryome” models of cross feeding support the concept that the cooperative production of essential nutrients maintains gut microbiota–host homeostasis [43]. In this regard, dysbiosis (maladaptation) may be characterized by bioenergetic failure, which results in the disappearance of cooperative hub taxa and the development of certain diseases [43]. Importantly, thanks to the Picrust2 analysis and the study of the Metacyc pathways, we found a gradual decrease in porphyrin metabolism and heme biosynthesis after beer consumption, according to the content of phenolic compounds, with the deepest decrease in dark beer (higher polyphenol content). The different roles attributed to heme remain controversial. On the one hand, heme has essential functions, such as being a key constituent of hemoproteins, redox enzymes and cytochromes [44,45]. On the other hand, free heme has pro-oxidant and pro-inflammatory effects [44,45]. Thus, porphyrin metabolism has been shown to be overactivated in obesity, a condition which is often accompanied by a pro-oxidative and pro-inflammatory state [46,47]. Moreover, heme oxidative effects may promote gut dysbiosis and gut epithelium damage [48,49]. Of note, heme synthesis and acquisition are important mechanisms developed by bacterial pathogens [50]. Because some *Streptococcus* species do not synthetize heme [43], we postulate that the observed increase in the relative abundance of *Streptococcus* after dark beer intervention may be related to the downregulation of heme biosynthesis. In light of the above, we hypothesize that the antioxidant effects of polyphenols may be driven, in part, by the downregulation of microbial pathways related to heme biosynthesis, which could ultimately result in reduced oxidative stress and inflammation. Further studies in this area are needed to unravel the role of bioenergetic-related pathways in metabolic diseases. 

In spite of the main strength of this study, which is its design, since, to our knowledge, this is the first clinical trial evaluating the impact of beers with different phenolic contents on the gut microbiota, this study has some limitations. First of all, the study population comprised healthy volunteers/patients with metabolic syndrome between 30 and 60 years old; therefore, our results cannot be extrapolated to other populations. Based on the limited sample size and duration of the intervention, these results should be considered cautiously. On the other hand, the lack of an exhaustive qualitative and quantitative analysis of the phenolic content limits the range of our results, as the intake of different beers might result in different outcomes. Finally, although Picrust2 analysis provides with rich information, it should be taken as a hypothesis generator, which should be further validated in coming studies. However, we strongly believe that these new findings could initiate new scientific debates about the consumption of beer and its repercussions in human health, and that these results will ensure further devoted trials to confirm these conclusions and new hypothesis derived from them.

## 5. Conclusions

In conclusion, we showed that gut microbiota composition may be affected by the moderate consumption of beer, and these changes seem to be conditioned by the metabolic status and polyphenol content in beer. Some of the observed changes may be driven by the antioxidant effects of polyphenols, which could be potentiated by some positive shifts in gut microbiota. Thus, our research provides evidence regarding the important regulatory functions of gut microbiota, which may mediate changes related to metabolic parameters induced by beer consumption and deserves further investigation to further analyze these results.

## Figures and Tables

**Table 1:** Clinical, anthropometric and biochemical characteristics of healthy volunteers and patients with metabolic syndrome.

Healthy Volunteers						Patients with Metabolic Syndrome				
	Washout	Dark	Lager	Alcohol-Free	*p*	Washout	Dark	Lager	Alcohol-Free	*p*
Weight (kg)	65.6 ± 7.75 *	65.66 ± 7.33 *	65.46 ± 7.69 *	65.99 ± 7.74 *	0.358	93.73 ± 14.41	93.25 ± 14.40	93.25 ± 15.39	93.84 ± 14.34	0.564
BMI (kg/m^2^)	22.85 ± 2.51 *	23.19 ± 3.08 *	23.14 ± 3.19 *	22.99 ± 2.52 *	0.642	34.58 ± 4.84	34.40 ± 4.76	34.37 ± 4.98	34.62 ± 4.73	0.564
Waist (cm)	80.3 ± 8.92 *	79.55 ± 7.94 *	81.25 ± 8.31 *	80.15 ± 7.58 *	0.377	109.06 ± 10.17	108.6 ± 12.02	104.3 ± 8.75	106.1 ± 7.48	0.247
Hip (cm)	97 ± 6.42 *	96.45 ± 6.39 *	96.9 ± 6.50 *	96.7 ± 6.39 *	0.597	114.94 ± 12.82	111.6 ± 11.63	111.3 ± 12.34	114.2 ± 11.03	0.077
SBP (mmHg)	114 ± 14.54 *	110.9 ± 14.77 *	115.15 ± 15.52 *	109.94 ± 9.44 *	0.325	137.45 ± 13.18	127.65 ± 9.82	130.75 ± 16.24	126.1 ± 10.48	0.216
DBP(mmHg)	69.35 ± 8.30 *	67.75 ± 9.73 *	71.5 ± 10.76 *	69.83 ± 7.22 *	0.633	88.8 ± 10.34	83.15 ± 7.48	86.45 ± 7.86	83.1 ± 7.68	0.298
Glucose (mg/dL	82.6 ± 6.75 *	86.3 ± 5.98 *	86 ± 8.99 *	84 ± 4.26 *	0.156	114.7 ± 36.24	112.6 ± 16.28	113.1 ± 25.45	109.7 ± 14.17	0.965
Urea (mg/dL)	28.6 ± 8.79	32.6 ± 8.94	29.9 ± 7.01	31.8 ± 10.10	0.605	32.3 ± 5.39	34.78 ± 5.84	34.6 ± 4.22	34.67 ± 5.36	0.386
Creatinine (mg/dL)	0.80 ± 0.13	0.83 ± 0.13	0.80 ± 0.14	0.79 ± 0.12	0.690	0.728 ± 0.19	0.7463 ± 0.20	0.781 ± 0.19	0.7211 ± 0.20	0.298
Uric Acid (mg/dL)	4.22 ± 0.79	4.57 ± 1.05	4.46 ± 0.89 *	4.41 ± 1.01 *	0.304	5.31 ± 1.42	5.81 ± 1.31	5.8778 ± 1.55	5.69 ± 1.33	0.036
Cholesterol (mg/dL)	192.5 ± 31.03	192.2 ± 31.31	191.8 ± 37.25	185.8 ± 28.57	0.901	208.6 ± 43.14	214.9 ± 41.66	204.8 ± 42.53	208.6 ± 48.88	0.782
HDL-c (mg/dL)	64 ± 12.56 *a	66.6 ± 13.74 *b	65.5 ± 13.38 *b	61.5 ± 12.32 *ab	0.039	48.7 ± 11.69	46.67 ± 11.05	46.7 ± 11.78	44.6 ± 11.60	0.173
LDL-c (mg/dL)	113.6 ± 28.57	111.6 ± 28.42	111.7 ± 35.99	108.7 ± 27.51	0.564	129.6 ± 31.18	133.1 ± 38.41	128.9 ± 34.51	124.1 ± 48.36	0.129
Triglycerides(mg/dL)	74.6 ± 38.62 *	69.9 ± 32.68 *	73.3 ± 29.55 *	77.5 ± 28.68 *	0.484	151.7 ± 89.16	201.4 ± 133.38	174.5 ± 114.91	212.3 ± 175.55	0.065
ALT (U/L)	20.4 ± 10.59 *	18.1 ± 6.74 *	15.9 ± 11.65 *	18.9 ± 8.87 *	0.086	38.2 ± 12.56	34.4 ± 10.92	31.2 ± 11.65	38.2 ± 12.61	0.035
AP (U/L)	53.9 ± 9.37 *	54.11 ± 7.04 *	54.44 ± 9.09 *	53.8 ± 8.10	0.990	69.8 ± 23.05	72.56 ± 18.55	68 ± 23.82	72.67 ± 27.49	0.455
HbA1c (%)	5.45 ± 0.20 *	5.39 ± 0.22 *	5.46 ± 0.20 *	5.4 ± 0.249 *	0.442	6.27 ± 0.65 c	6.06 ± 0.73 a	6.12 ± 0.68 ab	6.18 ± 0.73 b	0.002
Ferritin (ng/mL)	79.7 ± 49.72	66.6 ± 41.88	74.11 ± 50.16	73.61 ± 44.43	0.147	105.68 ± 90.59	102.42 ± 109.43	102.54 ± 91.97	97.28 ± 94.24	0.275
CRP (mg/L)	3.64 ± 2.82	6.22± 9.95	3.12 ± 3.34	2.24 ± 1.51	0.857	9.79 ± 10.24	8.14 ± 11.12	6.62 ± 6.18	5.525 ± 4.64	0.572
Insulin (µUI/mL)	5.78 ± 3.85 *	5.84 ± 3.33 *	5.63 ± 2.59 *	5.51 ± 3.063 *	0.392	20.03 ± 11.45	24.25 ± 20.03	19.02 ± 15.90	21.93 ± 19.51	0.197
C peptide (ng/mL)	0.89 ± 0.48 *	0.88 ± 0.35 *	0.94 ± 0.40 *	0.97 ± 0.41 *	0.426	2.50 ± 1.01	2.65 ± 1.41	2.58 ± 1.85	2.55 ± 1.41	0.208
HOMA-IR	1.19 ± 0.81 *	1.25 ± 0.76 *	1.22 ± 0.67 *	1.15 ± 0.65 *	0.207	5.8407 ± 4.01	7.03 ± 6.36	5.81 ± 5.78	6.38 ± 6.77	0.373
Vitamin D (ng/mL)	16.6 ± 7.96	20.89 ± 6.92	19.29 ± 9.42	20.59 ± 14.14	0.692	16.26 ± 4.47	19.28 ± 7.61	22.2 ± 10.37	18.11 ± 4.80	0.116

Values are presented as mean ± SD (standard deviation). *p* values were calculated for differences between groups using a Friedman test, considering *p* < 0.05 significant. Means denoted by a different letter indicate significant differences between groups (*p* < 0.05). * Denotes differences between healthy volunteers and subjects with metabolic syndrome. BMI: body mass index; SBP: systolic blood pressure; DBP: diastolic blood pressure; HDL-c: high-density lipoprotein cholesterol; LDL-c: low-density lipoprotein cholesterol; AL: alanine aminotransferase; AP: alkaline phosphatase; HbA1c: glycosylated hemoglobin; CRP: C-reactive protein; HOMA-IR: homeostatic model assessment of insulin resistance.

## Data Availability

The data are contained within the article and Supplementary Materials.

## Supplementary Materials

The following supporting information can be downloaded at: https://www.mdpi.com/article/10.3390/antiox11040696/s1, Figure S1: Taxon that changed significantly in each intervention period with respect to the washout period (baseline) at different taxonomic levels (DesSeq2 test). (a) Dark beer period; (b) lager beer period; (c) non-alcoholic beer period; Table S1: Dietary assessment according to total energy and macronutrients/fiber intake of healthy volunteers and participants with metabolic syndrome; Table S2: Metacyc pathways evaluated after each intervention period. Table S3: Common and different Metacyc pathways in the evaluated intervention periods.
