# Supplementary material for: Effect of Moderate Consumption of Different Phenolic-Content Beers on the Human Gut Microbiota Composition: A Randomized Crossover Trial
Source: Antioxidants (Basel). 2022 Mar 31;11(4):696. doi: 10.3390/antiox11040696 (PMC9027304; doi:10.3390/antiox11040696)

Supplemental Figure S1. Taxa that significantly changed between each intervention period with respect to the washout period (baseline) at different taxonomic levels: phylum, family and genus (DesSeq2 test; p<0.05). Values are presented as mean ±SD (standard deviation). a) Dark beer period; b) Lager beer period; c) without alcohol beer period.

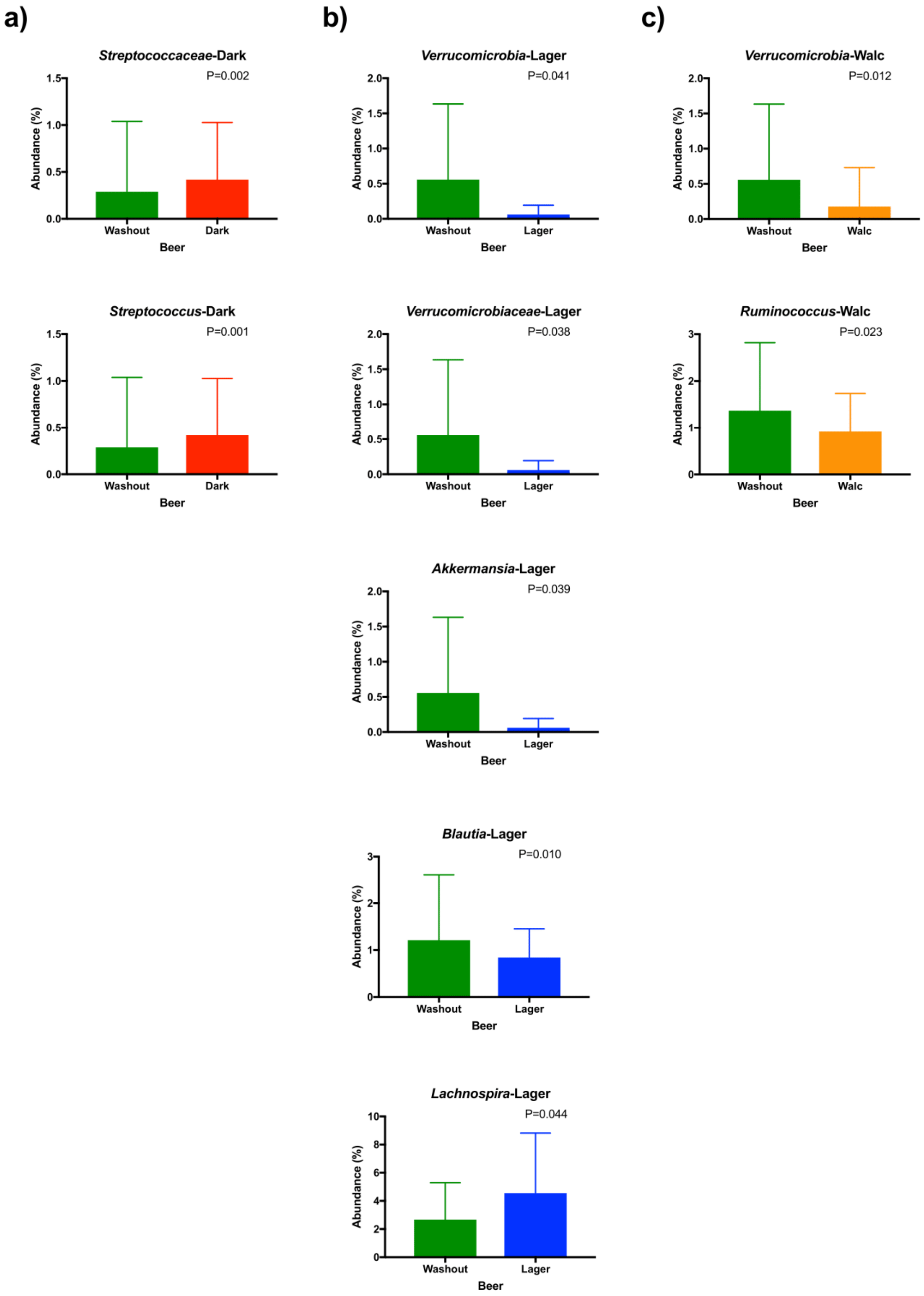

Supplement: Supplementary file 1 [file antioxidants-11-00696-s001.zip › antioxidants-1618343-supplementary/antioxidants-1618343-conversion supple/Supplemental figure S1.pdf]
