# Supplementary material for: Effect of Moderate Consumption of Different Phenolic-Content Beers on the Human Gut Microbiota Composition: A Randomized Crossover Trial
Source: Antioxidants (Basel). 2022 Mar 31;11(4):696. doi: 10.3390/antiox11040696 (PMC9027304; doi:10.3390/antiox11040696)
Supplement: Supplementary file 1 [file antioxidants-11-00696-s001.zip › antioxidants-1618343-supplementary/antioxidants-1618343-conversion supple/Supplemental Table S1.pdf]

Supplemental Table S1. Dietary assessment according to total energy and macronutrients/fiber intake of healthy volunteers and participants with metabolic syndrome

	Healthy volunteers				Subjects with metabolic syndrome				P	
	**Baseline**	**Alcohol-free**	**Lager**	**Dark**	**Baseline**	**Alcohol-free**	**Lager**	**Dark**	**Group**	**Beer**
**Total energy (kcal d-1)**	1610.06 ± 291.55	1760.06 ± 666.04	1581.93 ± 222.39	1612.59 ± 283.25	1617.20 ± 549.32	1585.49 ± 465.71	1462.45 ± 378.86	1629.25 ± 468.66	0.653	0.685
**Proteins (g d-1)**	65.68 ± 15.21	85.29 ± 61.13	69.71 ± 9.65	66.71 ± 15.79	70.96 ± 33.95	75.77 ± 17.63	70.32 ± 17.70	68.21 ± 13.90	0.943	0.353
**Lipids (g d-1)**	81.71 ± 24.43	84.96 ± 33.79	82.47 ± 15.23	81.28 ± 23.49	82.42 ± 38.71	85.46 ± 28.68	79.65 ± 23.27	88.60 ± 32.72	0.938	0.978
**Carbohydrates (g d-1)**	155.30 ± 26.83	162.97 ± 48.59	140.89 ± 35.95	154.93 ± 24.99	147.85 ± 44.81	127.66 ± 44.34	120.57 ± 29.30	138.13 ± 37.36	0.063	0.271
**Simple sugars (g d-1)**	68.26 ± 24.38	66.56 ± 28.27$	55.37 ± 12.03$	60.87 ± 17.55	52.68 ± 30.37	36.97 ± 12.31$	33.07 ± 16.07$	43.36 ± 27.22	0.006	0.129
**Dietetic fiber (g d-1)**	16.64 ± 6.40	16.41 ± 11.87	12.71 ± 3.57	14.80 ± 6.18	14.59 ± 4.97	15.67 ± 9.55	12.96 ± 5.54	14.46 ± 6.40	0.659	0.448

Values are presented as mean ±SD (standard deviation). $ Statistically significant difefferences (P<0.05) between groups (healthy volunteers and subjects with metabolic syndrome) according to each study intervention.
