# Supplementary material for: Effect of Moderate Consumption of Different Phenolic-Content Beers on the Human Gut Microbiota Composition: A Randomized Crossover Trial
Source: Antioxidants (Basel). 2022 Mar 31;11(4):696. doi: 10.3390/antiox11040696 (PMC9027304; doi:10.3390/antiox11040696)
Supplement: Supplementary file 1 [file antioxidants-11-00696-s001.zip › antioxidants-1618343-supplementary/antioxidants-1618343-conversion supple/Supplemental Table S2 .pdf]

Supplemental Table S2. Metacyc pathways evaluated after each intervention period

Abbreviation	Pathway
P101-PWY	ectoine biosynthesis
POLYAMSYN-PWY	superpathway of polyamine biosynthesis I
HOMOSER-METSYN-PWY	L-methionine biosynthesis I
PWY-181	photorespiration
PWY-5347	superpathway of L-methionine biosynthesis (transsulfuration)
PWY-7528	L-methionine salvage cycle I (bacteria and plants)
PWY-7315	dTDP-N-acetylthomosamine biosynthesis
PWY-7347	sucrose biosynthesis III
SUCSYN-PWY	sucrose biosynthesis I (from photosynthesis)
P241-PWY	coenzyme B biosynthesis
P261-PWY	coenzyme M biosynthesis I
PWY-5855	ubiquinol-7 biosynthesis (early decarboxylation)
PWY-5856	ubiquinol-9 biosynthesis (early decarboxylation)
PWY-5857	ubiquinol-10 biosynthesis (early decarboxylation)
PWY-6708	ubiquinol-8 biosynthesis (early decarboxylation)
UBISYN-PWY	superpathway of ubiquinol-8 biosynthesis (early decarboxylation)
PWY-6151	S-adenosyl-L-methionine salvage I
1CMET2-PWY	folate transformations III (E. coli)
PWY-1422	vitamin E biosynthesis (tocopherols)
PWY-6891	thiazole component of thiamine diphosphate biosynthesis II
PWY-7094	fatty acid salvage
PPGPPMET-PWY	ppGpp metabolism
PWY-7234	inosine-5'-phosphate biosynthesis III
PWY-7184	pyrimidine deoxyribonucleotides de novo biosynthesis I
PWY-7211	superpathway of pyrimidine deoxyribonucleotides <i>de novo</i> biosynthesis
PWY-6660	2-heptyl-3-hydroxy-4(1H)-quinolone biosynthesis
PWY-6662	superpathway of quinolone and alkylquinolone biosynthesis
PWY-7022	paromamine biosynthesis II
PWY-5188	tetrapyrrole biosynthesis I (from glutamate)
PWY-5189	tetrapyrrole biosynthesis II (from glycine)
CHLOROPHYLL-SYN	3,8-divinyl-chlorophyllide <i>a</i> biosynthesis I (aerobic, light-dependent)
HEME-BIOSYNTHESIS-II	heme b biosynthesis I (aerobic)
HEMESYN2-PWY	heme b biosynthesis II (oxygen-independent)
PWY-5531	3,8-divinyl-chlorophyllide a biosynthesis II (anaerobic)
PWY-7159	3,8-divinyl-chlorophyllide a biosynthesis III (aerobic, light independent)

PWY-5529	superpathway of bacteriochlorophyll a biosynthesis
PWY-5918	superpathway of heme b biosynthesis from glutamate
PWY-5920	superpathway of heme b biosynthesis from glycine
PWY0-1415	superpathway of heme b biosynthesis from uroporphyrinogen-III
PWY-5705	allantoin degradation to glyoxylate III
LEU-DEG2-PWY	L-leucine degradation I
PWY-5028	L-histidine degradation II
TYRFUMCAT-PWY	L-tyrosine degradation I
PWY-6185	4-methylcatechol degradation (ortho cleavage)
CATECHOL-ORTHO-CLEAVAGE-PWY	catechol degradation to β-ketoadipate
PWY-5415	catechol degradation I (meta-cleavage pathway)
PWY-5417	catechol degradation III (ortho-cleavage pathway)
PWY-5431	aromatic compounds degradation via β-ketoadipate
PWY-6957	mandelate degradation to acetyl-CoA
PWY-5179	toluene degradation V (aerobic) (via toluene-cis-diol)
PWY-5180	toluene degradation I (aerobic) (via o-cresol)
P23-PWY	reductive TCA cycle I
PWY-5392	reductive TCA cycle II
PWY-6992	1,5-anhydrofructose degradation
NPGLUCAT-PWY	Entner-Doudoroff pathway II (non-phosphorylative)
PWY-6713	L-rhamnose degradation II
PWY-6946	cholesterol degradation to androstenedione II (cholesterol dehydrogenase)
PWY-6641	superpathway of sulfolactate degradation
SALVADEHYPOX-PWY	adenosine nucleotides degradation II
PWY-6731	starch degradation III
PWY-7046	4-coumarate degradation (anaerobic)
P562-PWY	<I>myo</I>-inositol degradation I
PWY0-1261	anhydromuropeptides recycling I
PWY-5741	ethylmalonyl-CoA pathway
PWY-6876	isopropanol biosynthesis (engineered)
PWY-3781	aerobic respiration I (cytochrome c)
FERMENTATION-PWY	mixed acid fermentation
PWY-7254	TCA cycle VII (acetate-producers)
MET-SAM-PWY	superpathway of S-adenosyl-L-methionine biosynthesis
PWY0-781	aspartate superpathway
