# Supplementary material for: Effect of Moderate Consumption of Different Phenolic-Content Beers on the Human Gut Microbiota Composition: A Randomized Crossover Trial
Source: Antioxidants (Basel). 2022 Mar 31;11(4):696. doi: 10.3390/antiox11040696 (PMC9027304; doi:10.3390/antiox11040696)
Supplement: Supplementary file 1 [file antioxidants-11-00696-s001.zip › antioxidants-1618343-supplementary/antioxidants-1618343-conversion supple/Supplemental Table S3.pdf]

Supplemental Table S3. Common and different Metacyc pathways in the evaluated intervention periods.

**5 common elements in "Dark", "Lager" and "W-alc":**
CHLOROPHYLL-SYN: 3,8-divinyl-chlorophyllide *a* biosynthesis I (aerobic, light-dependent)
P562-PWY: *myo*-inositol degradation I
PWY-5531: 3,8-divinyl-chlorophyllide a biosynthesis II (anaerobic)
PWY-7159: 3,8-divinyl-chlorophyllide a biosynthesis III (aerobic, light independent)
PWY-7234: inosine-5'-phosphate biosynthesis III
**10 common elements in "Dark" and "Lager":**
HEME-BIOSYNTHESIS-II: heme b biosynthesis I (aerobic)
HEMESYN2-PWY: heme b biosynthesis II (oxygen-independent)
PWY-5529: superpathway of bacteriochlorophyll a biosynthesis
PWY-5918: superpathway of heme b biosynthesis from glutamate
PWY-5920: superpathway of heme b biosynthesis from glycine
PWY-7254: TCA cycle VII (acetate-producers)
PWY-7347: sucrose biosynthesis III
PWY-7528: L-methionine salvage cycle I (bacteria and plants)
PWY0-1261: anhydromuropeptides recycling I
SUCSYN-PWY: sucrose biosynthesis I (from photosynthesis)
**1 common element in "Lager" and "W-alc":**
PWY-6731: starch degradation III
**3 elements included exclusively in "Lager":**
PWY-1422: vitamin E biosynthesis (tocopherols)
PWY-7022: paromamine biosynthesis II
PWY-7046: 4-coumarate degradation (anaerobic)
**9 elements included exclusively in "W-alc":**
1CMET2-PWY: folate transformations III (E. coli)
FERMENTATION-PWY: mixed acid fermentation
HOMOSER-METSYN-PWY: L-methionine biosynthesis I
MET-SAM-PWY: superpathway of S-adenosyl-L-methionine biosynthesis
NPGLUCAT-PWY: Entner-Doudoroff pathway II (non-phosphorylative)
PWY-5347: superpathway of L-methionine biosynthesis (transsulfuration)
PWY-6151: S-adenosyl-L-methionine salvage I
PWY-6891: thiazole component of thiamine diphosphate biosynthesis II
PWY-7315: dTDP-N-acetylthomosamine biosynthesis
**45 elements included exclusively in "Dark":**

ARG+POLYAMINE-SYN: superpathway of arginine and polyamine biosynthesis
ARGORNPROST-PWY: L-arginine degradation (Stickland reaction)
CATECHOL-ORTHO-CLEAVAGE-PWY: catechol degradation to β-ketoadipate
LEU-DEG2-PWY: L-leucine degradation I
P101-PWY: ectoine biosynthesis
P23-PWY: reductive TCA cycle I
P241-PWY: coenzyme B biosynthesis
P261-PWY: coenzyme M biosynthesis I
POLYAMSYN-PWY: superpathway of polyamine biosynthesis I
PPGPPMET-PWY: ppGpp metabolism
PWY-181: photorespiration
PWY-3781: aerobic respiration I (cytochrome c)
PWY-5028: L-histidine degradation II
PWY-5179: toluene degradation V (aerobic) (via toluene-cis-diol)
PWY-5180: toluene degradation I (aerobic) (via o-cresol)
PWY-5182: toluene degradation II (aerobic) (via 4-methylcatechol)
PWY-5188: tetrapyrrole biosynthesis I (from glutamate)
PWY-5189: tetrapyrrole biosynthesis II (from glycine)
PWY-5392: reductive TCA cycle II
PWY-5415: catechol degradation I (meta-cleavage pathway)
PWY-5417: catechol degradation III (ortho-cleavage pathway)
PWY-5431: aromatic compounds degradation via β-ketoadipate
PWY-5705: allantoin degradation to glyoxylate III
PWY-5741: ethylmalonyl-CoA pathway
PWY-5855: ubiquinol-7 biosynthesis (early decarboxylation)
PWY-5856: ubiquinol-9 biosynthesis (early decarboxylation)
PWY-5857: ubiquinol-10 biosynthesis (early decarboxylation)
PWY-6185: 4-methylcatechol degradation (ortho cleavage)
PWY-6641: superpathway of sulfolactate degradation
PWY-6660: 2-heptyl-3-hydroxy-4(1H)-quinolone biosynthesis
PWY-6662: superpathway of quinolone and alkylquinolone biosynthesis
PWY-6708: ubiquinol-8 biosynthesis (early decarboxylation)
PWY-6713: L-rhamnose degradation II
PWY-6876: isopropanol biosynthesis (engineered)
PWY-6946: cholesterol degradation to androstenedione II (cholesterol dehydrogenase)
PWY-6957: mandelate degradation to acetyl-CoA
PWY-6992: 1,5-anhydrofructose degradation
PWY-7094: fatty acid salvage
PWY-7184: pyrimidine deoxyribonucleotides de novo biosynthesis I
PWY-7211: superpathway of pyrimidine deoxyribonucleotides *de novo* biosynthesis
PWY0-1415: superpathway of heme b biosynthesis from uroporphyrinogen-III

PWY0-781: aspartate superpathway
SALVADEHYPOX-PWY: adenosine nucleotides degradation II
TYRFUMCAT-PWY: L-tyrosine degradation I
UBISYN-PWY: superpathway of ubiquinol-8 biosynthesis (early decarboxylation)
